# Classification of surgical causes of and approaches to the chronically failing ileoanal pouch

**DOI:** 10.1007/s10151-022-02688-9

**Published:** 2022-08-30

**Authors:** E. M. Meima-van Praag, M. A. Reijntjes, R. Hompes, C. J. Buskens, M. Duijvestein, W. A. Bemelman

**Affiliations:** 1 Department of Surgery, Amsterdam Gastroenterology Endocrinology and Metabolism, Amsterdam UMC, University of Amsterdam, Meibergdreef 9, 1105 AZ Amsterdam, The Netherlands; 2 Department of Gastroenterology and Hepatology, Amsterdam Gastroenterology Endocrinology and Metabolism, Amsterdam UMC, University of Amsterdam, Meibergdreef 9, Amsterdam, The Netherlands; 3 IBD Unit, Gastroenterology and Endoscopy, IRCCS Ospedale San Raffaele and University Vita-Salute San Raffaele, Milan, Italy

**Keywords:** Ileoanal pouch surgery, Redo surgery, Chronically failing pouch, TAMIS

## Abstract

**Background:**

Although there are various surgical causes of and therapeutic approaches to the chronically failing ileoanal pouch (PF), cases are often detailed without distinguishing the exact cause and corresponding treatment. The aim of our study was to classify causes of PF and corresponding surgical treatment options, and to establish efficacy of surgical approach per cause.

**Methods:**

This retrospective study included all consecutive adult patients with chronic PF surgically treated at our tertiary hospital between July 2014 and March 2021. Patients were classified according to a proposed sub-classification for surgical related chronic PF. Results were reported accordingly.

**Results:**

A total of 59 procedures were completed in 50 patients (64% male, median age 45 years [IQR 34.5–54.3]) for chronic PF. Most patients had refractory ulcerative colitis as indication for their restorative proctocolectomy (68%). All patients could be categorized according to the sub-classification. Reasons for chronic PF were septic complications (*n* = 25), pouch body complications (*n* = 12), outlet problems (*n* = 11), cuff problems (*n* = 8), retained rectum (*n* = 2), and inlet problems (*n* = 1). For these indications, 17 pouches were excised, 10 pouch reconstructions were performed, and 32 pouch revision procedures were performed. The various procedures had different complication rates. Technical success rates of redo surgery for the different causes varied from 0 to 100%, with a 75% success rate for septic causes.

**Conclusions:**

Our sub-classification for chronic PF and corresponding treatments is suitable for all included patients. Outcomes varied between causes and subsequent management. Chronic PF was predominantly caused by septic complications with redo surgery achieving a 75% technical success rate.

## Introduction

Restorative ileal pouch-anal anastomosis (IPAA) after proctocolectomy is a well-established procedure often used in patients with ulcerative colitis or familial adenomatous polyposis. Functional outcomes are often excellent for the majority of patients, although a significant number of pouches do fail over time [1]. A chronically failing pouch (PF) can be caused by various conditions e.g. chronic pelvic sepsis, mechanical in- or outlet problems, presence of Crohn’s disease (CD), perianal fistulas and refractory chronic pouchitis or cuffiti*s*. The incidence of PF is reported to be up to 7 and 9% after 3 and 5 years, respectively [2], with pelvic sepsis and fistula as the leading indications for surgical correction [1]. Both surgical (chronic pelvic sepsis, mechanical problems) as well as medical (pouchitis, cuffitis, CD and/or fistula) causes of PF might eventually require redo pouch surgery [2].

Pouch redo procedures and excision aim to improve patient quality of life, while redo procedures have the additional objective of preserving bowel continuity. Both types of procedure are challenging and entail operating deep in the pelvis.. Most researchers describing redo surgery for PF combine all patients irrespective of cause and treatment approach. Studies report overall success rates of redo surgery for PF of approximately 70% [3–6]. Although these studies often clearly describe indications for surgical treatment, outcomes after redo surgery following PF are at best described according to septic and non-septic complications [7–11]. However, there is a general lack of detail as to the underlying etiology and how surgical approaches may vary according to this etiology. So far, a classification of exact types of chronic PF and the therapeutic options has not been proposed [3], probably because of the complexity of such a classification.

Indication and treatment classification enables reporting on success rates per PF cause and therapeutic intervention. This facilitates (shared) decision-making about the best option for the individual patient. Moreover, introduction of a sub-classification facilitates routine registration of chronic PF and redo-pouch procedures. The aim of our study was to classify causes of PF and corresponding surgical treatment options, and to establish the efficacy of surgical approach per cause.

## Materials and methods

### Study design and population

This retrospective observational cohort study included all consecutive adult patients that underwent IPAA surgery and developed chronic PF, surgically managed by laparoscopic and/or open surgery mostly in combination with TAMIS at our referral center. Patients undergoing redo surgery for all chronic PF indications between July 2014 and March 2021 in the Amsterdam UMC, location AMC, were included. These were patients who underwent initial IPAA surgery in the Amsterdam University Medical Centers (UMC), Academic Medical Center (AMC) location, as well as patients referred from elsewhere. Chronic PF was defined as a long-term (≥ 3 months) pouch-related complication (including pouch dysfunction) requiring pouch redo surgery or excision regardless of the surgical indication and functional outcome of the pouch (with or without permanent diversion by ileostomy). Pouch excision was defined as resection of the primary pouch with construction of a permanent ileostomy. For pouch redo surgery, a distinction was made between a pouch ‘reconstruction’ and pouch ‘revision’. Pouch ‘reconstruction’ was defined as resection of the primary pouch with construction of a new IPAA and ‘revision’ was any other surgical modification of the primary pouch, with or without disconnection of the anastomosis. Bowel continuity was pursued in both types of redo surgery. Early PF indications such as acute postoperative leakage or hemorrhage requiring redo surgery within 3 months after IPAA surgery are not included in this sub-classification. According to local law and the Medical Ethics Committee at the Amsterdam UMC, ethical approval was waived for this retrospective study. All eligible patients received an opt-out letter and could withdraw permission to collect data for this study.

### Surgical procedure and classification

**Fig. 1:**
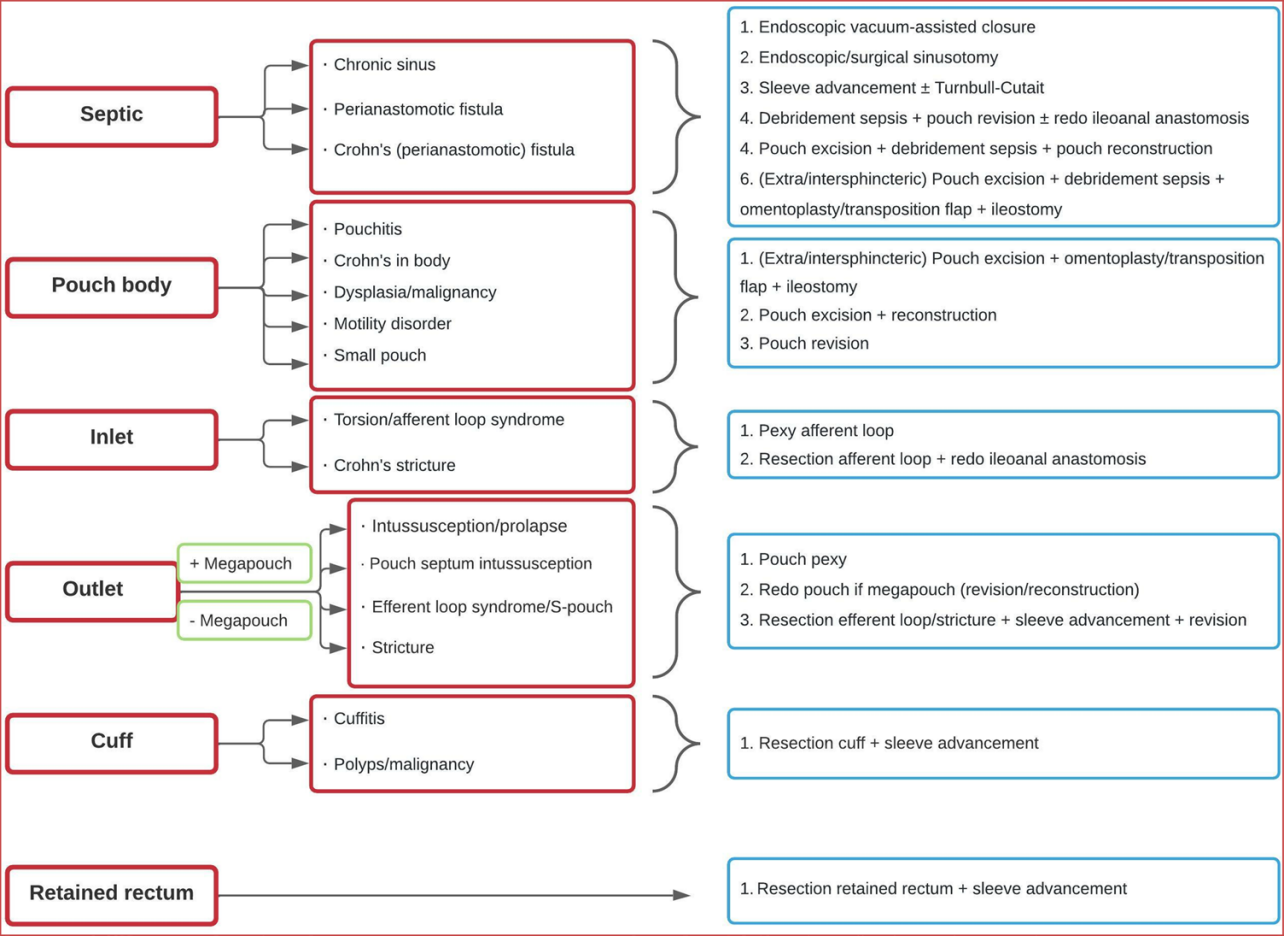
Sub-classification for the surgical related chronically failing pouch [27–29]

Redo surgery for PF was performed either completely transanally via transanal minimally invasive surgery (TAMIS [12]), transabdominally (open/laparoscopic) or combined transanally and transabdominally. The preferred surgical approach per procedure in the Amsterdam UMC, location AMC, is summarized in Table 1*.* A classification for surgical related chronic PF was developed categorizing different types of PF and subsequent surgical management options to overcome random classification of chronic PF (Fig. 1). In this classification, six main categories causing PF are presented in the left-hand column with corresponding sub-categories in the second column. The third column displays the various surgical management options for the various etiologies of chronic PF. CD in the pouch was either histopathologically proven or strongly suspected. Outlet problems were diagnosed by endoscopy or defecating pouchography where pressure resulted in a collapsed outlet, and were unresponsive to non-surgical treatment (biofeedback/catheterization/irrigation). A retained rectum was defined as residual rectal tissue, exceeding 2 cm proximal to the dentate line.

**Table 1:** Preferred approach of redo surgery types in Amsterdam UMC

Endoscopic vacuum-assisted surgical closure	Endoscopic + trans-anal closure
Endoscopic/surgical sinusotomy	Endoscopic/TAMIS
Pexy or resection afferent loop	Transabdominal
Pexy pouch	Transabdominal
Redo pouch (reconstruction/revision) ± debridement sepsis	TAMIS + transabdominal
Pouch/sleeve advancement ± Turnbull–Cutait	TAMIS ± transabdominal
Resection stricture/cuff/efferent loop/retained rectum	TAMIS ± transabdominal
Pouch excision + omentoplasty/transposition flaps ± debridement sepsis + ileostomy	TAMIS + transabdominal

*TAMIS* transanal minimally invasive surgery

### Demographic and outcome variables

Patient demographics, medical history, indications for surgery, perioperative and postoperative outcomes were retrieved from electronic patient records. Outcome variables included perioperative mortality, postoperative morbidity and permanent ileostomy presence. Postoperative morbidity was graded according to the Clavien–Dindo classification [13], and severe postoperative morbidity was defined as Clavien–Dindo grade ≥ IIIa. Short-term anastomotic leaks were radiologically, endoscopically or intraoperatively diagnosed within 3 months after redo surgery. In this study, functional success of redo surgery was defined as a redo procedure which resulted in a functional pouch at the end of follow-up without secondary redo surgery. Technical success of pouch redo surgery was defined as a redo procedure followed by an intact ileoanal anastomosis regardless the presence of a defunctioning ileostomy that was not (yet) closed.

### Statistical analysis

Data were collected and stored in an electronic database. All PF procedures were described according to the suggested sub-classification. Categorical data were presented as amount of redo procedures (*n*) and their proportion as a percentage. Continuous data were presented as mean and standard deviation (SD) or as median and interquartile range (IQR), according to their distribution. Analyses were performed using IBM^®^ SPSS^®^ for Windows^®^ version 26 (IBM Corp., Armonk, NY, USA).

## Results

### Baseline characteristics

A total of 59 surgical redo procedures in 50 patients were performed for chronic PF. The median follow-up after pouch-redo surgery was 19.0 (IQR 5.0–33.1) months*.* Patients were predominantly male (64.0%) with a median age of 45.0 (IQR 34.5–54.3) years. Initial indications for restorative proctocolectomy were predominantly medical refractory ulcerative colitis (68.0%) and familial adenomatous polyposis (24.0%). Further baseline characteristics are listed in Table 2.

**Table 2:** Baseline characteristics (*n* = 50 patients)

Male sex, *n* (%)	32 (64)
Age at redo surgery, years, median (IQR)	45.0 (34.5–54.3)
Initial IPAA indication, *n* (%)	
Ulcerative colitis Crohn’s colitis Familial adenomatous polyposis^a^ Carcinoma of the rectum and polyposis Other	34 (68) 1 (2) 12 (24) 1 (2) 2 (4)
ASA class, *n* (%)	
1 2 3	12 (24) 32 (64) 6 (12)
Body mass index (kg/m^2^) ≥ 30, *n* (%)	4 (8)
Current smoking, *n* (%)	12 (24)
Preoperative ileostomy, *n* (%)	24 (48)
Intraoperative use of steroids, *n* (%)	3 (6)
Comorbidity	
Diabetes mellitus, *n* (%) Ischemic heart disease, *n* (%)	3 (6) 5 (10)
Interval between initial IPAA and redo procedure, years, median (IQR)	9.0 (3.8–20.0)

*IPAA* ileal pouch-anal anastomosis, *ASA* American Society of Anesthesiologists, scored by an anesthesiologist, *n* number of patients

^a^Includes all types of hereditary polyposis coli

### Perioperative characteristics

#### Indications and surgical procedures

Indications for surgical treatment of chronic PF were septic complications (*n* = 25), pouch body complications (*n* = 12) e.g. complications of the pouch related to CD (*n* = 5) and chronic refractory pouchitis (*n* = 3), outlet problems (*n* = 11) with (*n* = 8) and without (*n* = 3) mega-pouch, cuff problems (*n* = 8), retained rectum (*n* = 2), and inlet problems (*n* = 1). Out of 59 surgical redo procedures, 16 involved a pouch excision and one a resection of the top of the pouch with creation of a definitive ileostomy. The remaining 42 surgical redo procedures involved 10 pouch reconstructions and 32 pouch revision procedures. The 59 procedures were performed in 50 patients, as 7 patients required 2 and 1 required 3 pouch redo procedures. An overview of the performed pouch excisions and redo surgeries per PF cause is provided in Table 3.

**Table 3:** Intra- and postoperative outcomes (*n* = 59 procedures)

Surgical procedure (*n*)	Surgical approach^a^	Anastomotic technique^a^	Postoperative complications (Clavien–Dindo ≥ 3)^a^	Anastomotic leakage^a^	Follow-up in months^b^
Overall (59)	TAMIS/Open: 38 (64%) TAMIS/lap: 12 (20%) TAMIS: 5 (8%) Laparoscopic: 3 (5%) Open: 1 (2%)	Manual: 34 (9%) Stapled: 2 (5%) TC: 2 (5%)	20 (34%)	11 (29%)	17 (3.0–32.0)
Pouch excision (17)	TAMIS/open: 10 (5%) TAMIS/lap: 6 (3%) Laparoscopic: 1 (5%)	n.a	1 (6%)	n.a	3.0 (1.5–13.5)
Pouch excision + (10) reconstruction	TAMIS/open: 9 (90%) Open: 1 (1%)	Manual: 8 (80%) Stapled: 1 (10%) TC: 1 (10%)	6 (60%)	2 (20%)	14.5 (1.0–22.0)
Pouch revision (32)	TAMIS/open: 19 (59%) TAMIS/lap: 6 (19%) TAMIS: 5 (16%) Laparoscopic: 2 (6%)	Manual: 26 (81%) Stapled: 1 (3%) TC: 1 (3%) n.a.: 4 (13%)	13 (41%)	9 (28%)	25.0 (9.3–35.8)

*TAMIS* transanal minimally invasive surgery, *Lap* laparoscopic, *TC* Turnbull–Cutait, *n.a.* not applicable, *n* amount of surgical procedures

^a^Values are *n* (percentage)

^b^Values are median (IQR)

### Intraoperative characteristics

Out of all 59 procedures, 49 were performed via a combined TAMIS and open (*n* = 37) or TAMIS and laparoscopic (*n* = 12) approach. Five procedures were performed via TAMIS only, 3 were performed laparoscopically and 2 consisted of an open abdominal approach. One of the pouch excision procedures required conversion from a combined TAMIS and laparoscopic to a combined TAMIS and open procedure due to severe adhesions. Thirty-five out of 39 (combined) open abdominal surgery procedures were performed via lower midline laparotomy and 4 via Pfannenstiel incision. Pouch excisions were either combined TAMIS and open (*n* = 10), TAMIS and laparoscopic (*n* = 5), laparoscopic (*n* = 1) or TAMIS (*n* = 1) procedures. The pelvic cavity was filled by pediculized omentoplasty in 14 patients (23.7%), and pouch mesentery was used in 3 (5.1%). In one patient, omento- and mesoplasty were combined and in another, the ileocolic mesentery was used to fill the pelvic cavity. An ileoanal anastomosis was created in 38 redo procedures, 35 (92.1%) of which were hand-sewn. One anastomosis was stapled, and in 2 procedures a Turnbull–Cutait was technique applied; the ileoanal anastomosis was therefore delayed until a second stage. Intraoperative characteristics are listed in Table 3.

### Postoperative course

Patients were discharged from the hospital at a median 6.0 (IQR 4.0–10.0) days after surgery. In 20 (33.8%) patients, severe postoperative complications (Clavien–Dindo ≥ 3) were reported. More than half of the reinterventions were indicated for anastomotic leak (11/20, 55.0%). Therefore, out of 38 ileoanal anastomoses that were created during redo surgery, 28.9% (*n* = 11) had a postoperative anastomotic leak. For acute anastomotic leak, 5 patients underwent a surgical reintervention, 4 patients underwent a combined surgical and endoscopic reintervention, 1 patient underwent a combined surgical and radiological reintervention, and 1 patient had an endoscopic reintervention. The reintervention for acute anastomotic leak was successful in 7/11 patients, which was not correlated to type of reintervention. Postoperative outcomes are shown in Table 3. At the end of follow-up, a total of 33 patients had a permanent ileostomy; 17 after pouch excision and 16 after pouch revision/reconstruction. However, one patient was still awaiting ileostomy closure after reconstruction and three patients had a permanent ileostomy despite technical success.

### Success rates of pouch redo surgery

Out of 50 patients, 12 underwent a pouch excision. The remaining 38 patients underwent redo surgery for chronic PF and 24 (63.2%) had a functional pouch at the end of follow-up. An intended temporary ileostomy after a redo procedure was never reversed in 1 patient despite technical success of the procedure. One patient with technical success proven by computed tomography scan and endoscopy of the pouch is still awaiting reversal of ileostomy created during the redo procedure. The technical success rate of the first redo procedure in this cohort is therefore 71.1% (27/38). After the first redo procedure, functional success was achieved in at least 25/38 patients (> 65.6%). The first pouch-redo procedure failed in the remaining 11 patients; 4 patients required pouch excision surgery with a permanent ileostomy and in 3 the loop ileostomy was converted into a permanent end-ileostomy. A second redo pouch surgery was performed in 4 patients and 3 were successful. An overview of the surgical redo procedures per indication with corresponding success rates is provider in Table 4. Overall technical success was achieved in 71.4% (30/42) of redo procedures, which was comparable among patients with (22/31, 70.1%) and without (8/11, 72.7%) a protecting ileostomy.

**Table 4:**
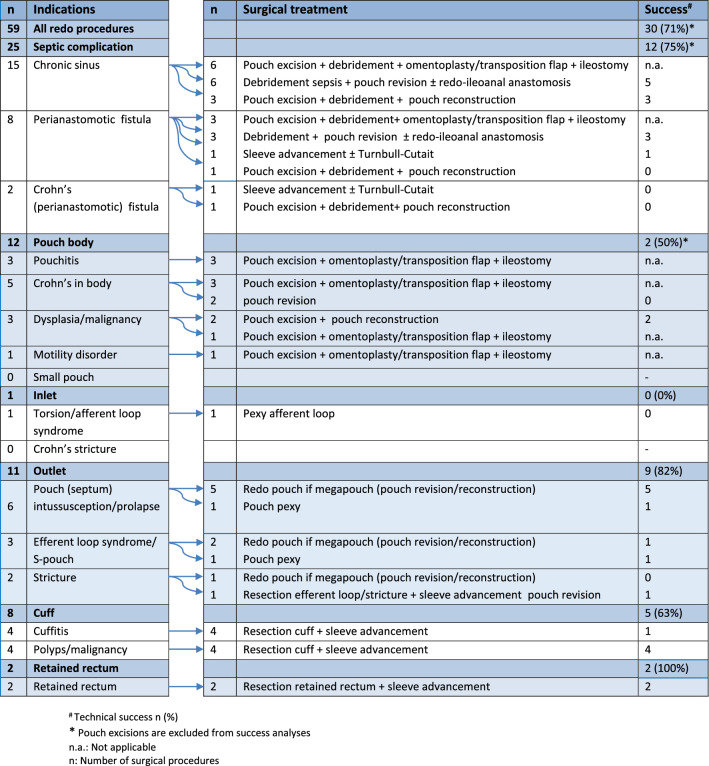
Amsterdam sub-classification for the chronically failing pouch applied on study cohort

### Septic complications

Twenty-five patients had a redo procedure due to septic conditions, of which 15 had a chronic sinus caused by a chronic leak. Four patients underwent immediate pouch excision for a chronic sinus, including the patient that had proctocolectomy because of presumed Crohn’s colitis. Five patients underwent a successful pouch revision after debridement of a chronic sinus; in 3 patients a pouch reconstruction was performed, with technical success in all. One of these patients is still awaiting ileostomy closure. One patient required pouch excision after a failed pouch revision for a chronic sinus and another required pouch excision for a septic anastomotic leak after a cuff excision for cuffitis. Eight patients had redo surgery for perianastomotic fistula; 3 had a pouch excision, 3 a pouch revision, 1 a sleeve advancement and 1 a pouch reconstruction. The patient who underwent a pouch reconstruction eventually required a permanent ileostomy due to a recurring fistula. Two patients had a Crohn’s related fistula; one underwent a sleeve advancement and in the other a pouch reconstruction was performed. Both procedures failed due to a recurring fistula. Patients were followed for a median time span of 18.1 (IQR 6.0–33.2) months after their redo procedure.

### Pouch body complications

Twelve patients had pouch body related problems. Three patients with chronic refractory pouchitis underwent a pouch excision. Three patients had CD of the pouch body, of which 1 had immediate pouch excision and 1 had a pouch excision after a failed pouch revision for CD. Another patient with CD of the pouch body had the top of the pouch resected with creation of a definitive ileostomy after 2 failed pouch revisions for CD (fistula), as mentioned above. Three patients had dysplasia in the pouch for which a pouch excision was performed in 1 patient and a successful redo pouch was made in 2. One patient with a motility disorder of the pouch underwent a pouch excision. Median length of follow-up of redo procedures was 21.2 (IQR 5.5–43.0) months.

### Inlet

One patient with a torsion of the afferent loop underwent pexy of the loop. After this procedure, this patient required a second pouch revision due to a perianastomotic fistula, which was previously described. This patient was followed for 50.3 months.

### Outlet

Six of the 11 patients with outlet problems had complicated evacuation of the pouch due to intussusception. Four patients had distal septa in the pouch that intussuscepted into the anus blocking evacuation during straining. One patient had pouch wall intussusception and another had both pouch wall- and septal intussusception. For septal intussusception, 2 patients underwent a successful pouch revision and 1 patient underwent a successful pouch reconstruction. One patient developed septal intussusception after failed redo surgery for efferent loop syndrome in an S-pouch for which a pouch revision was performed. Eventually, this patient underwent permanent ileostomy surgery despite technical success. For anterior pouch wall intussusception, 1 patient underwent successful pexy surgery. A pouch reconstruction was performed in 1 patient with both wall and septal intussusception followed by permanent ileostomy surgery despite technical success. Two out of 3 patients with efferent loop syndrome underwent a pouch revision. In 1 revision was successful and 1 required second redo surgery for septal intussusception, as previously mentioned. The third patient underwent a pouch revision for efferent loop syndrome after a cuff excision for cuffitis, which was technically successful. The patient desired a pouch excision and the creation of a Kock-pouch at a later stage [14]. One of the 2 patients with pouch stricture had a successful stricture excision. The other patient undergoing a pouch reconstruction required a permanent ileostomy for a postoperative chronic sinus. Patients were followed for a median time span of 24.8 (IQR 8.9–32.2) months after their redo procedures for outlet problems.

**Fig. 2:**
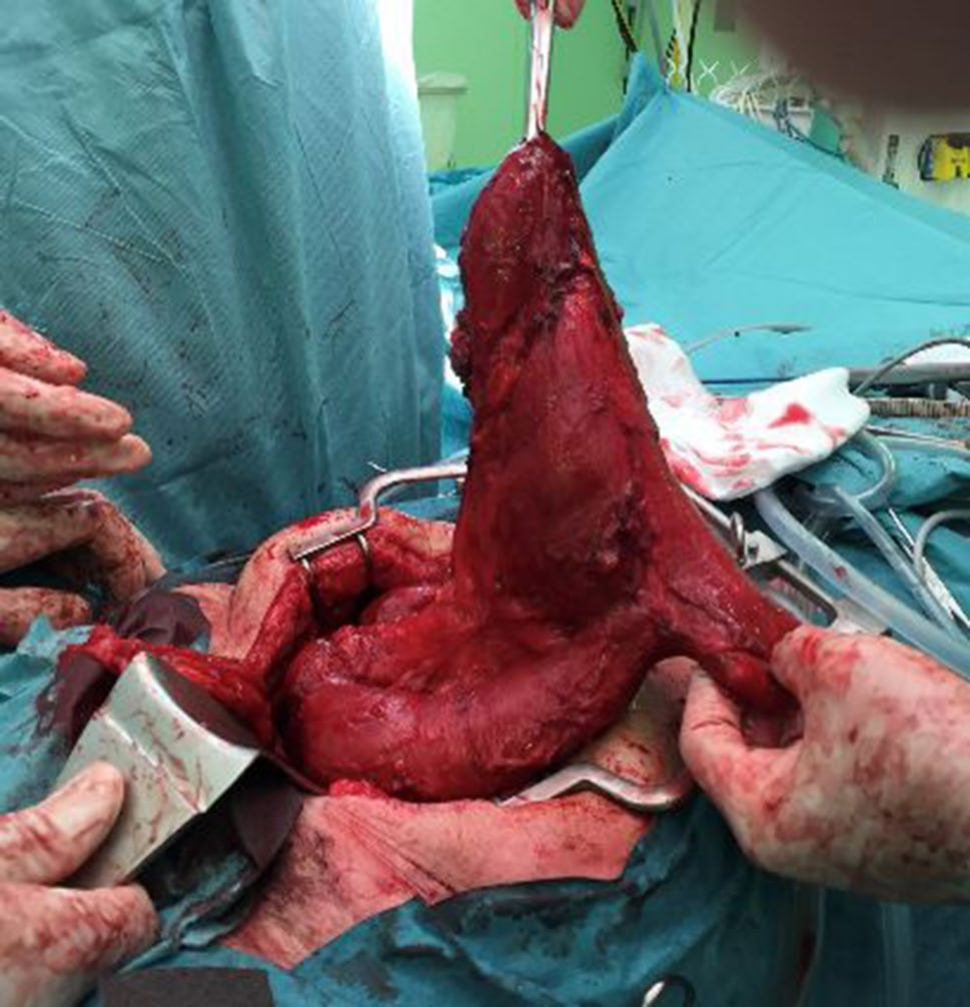
Excision of megapouch

Eight out of 11 patients with outlet problems had a megapouch (Fig. 2). Of these, a pouch revision was performed in five, a pouch reconstruction in two, and a pouch pexy performed in one. One of the pouch revisions failed and all other procedures in patients with a megapouch were technically successful.

### Cuff

Eight patients had cuff problems; 4 patients had refractory cuffitis and 4 had cuff dysplasia (polyps *n* = 3, malignancy *n* = 1). Median length of follow-up was 27.8 (15.5–40.7) months. All patients with cuffitis underwent cuff excision with sleeve advancement. One of these procedures was technically successful. For 2 patients, a pouch excision followed due to a chronic sinus (*n* = 1) and perianastomotic fistula (*n* = 1), as mentioned earlier. For 1 patient, second redo surgery for outlet complications followed, as mentioned earlier. All 4 patients with cuff dysplasia underwent cuff excisions with sleeve advancement, which were all technically successful.

### Retained rectum

Two patients with a retained rectum after IPAA surgery underwent a successful excision of the retained rectum followed by a new ileoanal anastomosis. Patients were followed for 8.8 and 32.2 months.

## Discussion

The proposed sub-classification of causes for PF and the subsequent treatment alternatives enabled the categorization of our patient cohort. This sub-classification supports continuity in surgical treatment for chronic failure of the ileoanal pouch. It furthermore supports routine registration of patients with chronic PF after IPAA surgery, thereby permitting reliable comparison of results with other research groups. Due to the sub-classification, success rates of redo surgery according to cause and management of chronic PF could be reported. Pouch redo procedures aiming at preservation of the continuity demonstrated acceptable technical success rates of 71.4%. Success rates of redo surgery for PF differed from 0 to 100% per surgical indication.

Most successful were redo procedures for retained rectum (100%), followed by redo for outlet disorders (82%, with 88% for megapouches) and chronic pelvic sepsis (75%). Cuff excision for refractory pouchitis had a low success rate of 25% as opposed to cuff excision for polyposis (75%). Management of pouch body problems for CD failed in all patients, as opposed to a 100% technical success rate in redo surgery for neoplastic lesions, although there were few patients with these conditions. The relative overrepresentation of patients with familial adenomatous polyposis in this cohort is due to the high amount of patients that were referred from other hospitals. Several complications mentioned in the sub-classification are rare and thus we were able to include only a small number of patients.

Postoperative complications including anastomotic defects and need for reintervention was common after redo surgery. In our cohort, severe postoperative complications occurred in one-third of patients and anastomotic leaks occurred after 28.9% of redo procedures, corresponding with available literature reporting anastomotic leak rates after redo surgery between 0 and 41% [9, 15–18]. The largest series on transabdominal redo pouch surgery to date had a high morbidity rate of 53%, including all types of postoperative complications [9]. Unfortunately the authors did not specify the complications according to the definition used in the current study (Clavien–Dindo ≥ 3). Studies assessing colorectal redo-procedures report severe postoperative complication rates between 26 and 32%, which is in line with the 33.8% reported in our study [15–20]. Anastomotic leak rates were lower in the study by Remzi et al., as the authors distinguished patients with short term pelvic sepsis from those with anastomotic dehiscence [9]. With our definition higher leak rates are obvious. Leaking anastomoses can be salvaged by applying endoscopic vacuum assisted surgical closure (EVAC), which is proven to be effective in preserving the ileoanal anastomosis [21].

With the introduction of TAMIS surgery, redo surgery is performed with increasing regularity and variety [4]. Most of the redo pouch surgeries at our unit were performed via combined transabdominal and TAMIS approach (55/59, 93.2%). An important advantage of the TAMIS approach includes a higher chance of preservation of the distal part of the pouch and surrounding vital structures. The improved visibility and access to the pelvis facilitates the complex redo surgery deep down in the pelvis [12, 22, 23]. Moreover, this technique enables a minimally invasive transabdominal approach in a great number of patients.

Series published on TAMIS surgery for redo procedures after IPAA predominantly focus on septic anastomotic complications [4, 24]. Although anastomotic complications account for the most important surgical causes of chronic PF [1], a number of mechanical and/or inflammatory complications must be managed surgically as well. Minimally invasive surgical approaches including TAMIS in pouch surgery show favorable functional outcomes [6, 25, 26]. As functional results were not investigated in our retrospective study, future prospective studies monitoring functional outcomes after redo surgery are warranted. Furthermore, details about primary IPAA were limited as numerous patients were referred to our tertiary center for surgical treatment for chronic PF.

Our study has some limitations The relatively small cohort is one of the main limitations. Some complications in the sub-classification such as inlet complications or retained rectum were too rare to draw conclusions about surgical success. As high volume centra could provide more patient data on this, classification of surgical treatment of PF remains essential. External validation from high volume centra for the proposed sub-classification for chronic PF and the suggested corresponding therapeutic approach is therefore essential. Adding a significant number of patients with a longer follow-up after surgery for PF will help enable us to draw more robust conclusions with respect to success rates of the management of the various types of chronic PF. Another limitation is that patients can have multiple chronic PF indications as described in the sub-classification. In that case, either an overlapping surgical treatment can be performed or a combination of (medical and) surgical treatments can be considered.

## Conclusions

With the proposed sub-classification, management of the complex problem of chronic PF becomes more comprehensible and makes results comparable. Our series showed that redo pouch procedures for chronic septic problems, outlet problems with or without megapouch, polyposis of the cuff and retained rectum all had favorable success rates, justifying the indication for redo pouch surgery. Conversely, the selected patients that had redo surgery for CD of the pouch, and for refractory cuffitis had unfavorable outcomes. It is, essential to combine good quality data from multiple units in order to draw more robust conclusions.
